# Relationship between cognitive function and functional outcomes in remitted major depression

**DOI:** 10.1186/s12888-024-05675-6

**Published:** 2024-04-24

**Authors:** Ruiqing Luo, Ningdan Fan, Yikai Dou, Yu Wang, Min Wang, Xiao Yang, Xiaohong Ma

**Affiliations:** 1 Mental health center and psychiatric laboratory, West China Hospital of Sichuan University, 610041 Chengdu, China; 2 Chongqing Mental Health Center, 400036 Chongqing, China

**Keywords:** Major depressive disorder, Cognitive function, Neurocognition, Functional outcome, Social function

## Abstract

**Background:**

Few studies have focused on functional impairment in depressed patients during symptomatic remission. The exact relationship between cognitive performance and functional outcomes of patients with Major depressive disorder (MDD) remains unclear.

**Methods:**

Participants diagnosed with MDD were included and interviewed at both baseline and follow-up. Cognitive function was assessed during acute episodes using the Cambridge Neuropsychological Test Automated Battery (CANTAB), which targeted attention (Rapid Visual Processing - RVP), visual memory (Pattern Recognition Memory - PRM), and executive function (Intra-Extra Dimensional Set Shift - IED). The 17-item Hamilton Depression Scale (HAMD) was used for symptom assessment. Participants were divided into two groups based on their SDSS (Social Disability Screening Schedule) scores, and the differences between their demographic information, HAMD scores, and baseline CANTAB test results were compared. Logistic regression analysis was used to identify cognitive predictors of social function during symptomatic remission.

**Results:**

According to the SDSS score at follow-up, 103 patients were divided into the normal social function group (*n* = 81,78.6%) and the poor social function group (*n* = 22, 21.4%) during clinical remission. Participants with poorer social function performed worse in the visual memory (PRM) and executive function tests (IED) at the baseline. Logistic regression analysis suggested that performance on the PRM (95%CI = 0.31–0.93, *p* = 0.030) and IED (95%CI = 1.01–1.13, *p* = 0.014) tests, instead of less severe symptoms, significantly contributed to functional outcomes.

**Conclusion:**

Better performance in visual memory and executive function during acute episodes may predict better social functional outcomes in individuals with MDD. A potential early intervention to improve social function in individuals with MDD could include the treatments for executive function and visual memory.

**Supplementary Information:**

The online version contains supplementary material available at 10.1186/s12888-024-05675-6.

## Introduction

Major depressive disorder (MDD) is a common mental illness characterized by anhedonia, low motivation, and cognitive deficits [1, 2]. It is currently a prominent public health concern and a leading cause of global mental health disability [3]. Individuals with MDD often exhibit social dysfunction, resulting in impairments in both occupational and relational aspects [4, 5]. These dysfunctions not only diminish the quality of life but also escalate societal costs [6, 7]. A recent study showed that workplace costs constitute 61% of the additional economic burden in adults with MDD, emphasizing the critical role of social function in MDD [8]. Early investigations have suggested a strong correlation between symptoms and social functioning [9, 10]. However, emerging data do not show a complete correspondence between functioning scales and symptoms [11]. Notably, social dysfunction is reported not only in the acute phase but also during symptomatic remission in patients with MDD. In a large study (Sequenced Treatment Alternatives to Relieve Depression), 20–40% of patients achieving symptom remission continue to experience ongoing functional impairment [12]. Nevertheless, few studies have focused on functional impairment in depressed patients during symptomatic remission, which poses challenges for early intervention. Thus, understanding the factors related to functional impairment in remitted MDD is a critical issue, addressing not only individual perspectives but also societal considerations.

Numerous studies have consistently shown that patients in the acute phase of a depressive episode exhibit cognitive impairment [2]. The Cambridge Neuropsychological Test Automated Battery (CANTAB) is a frequently used, well-validated tool for assessing multi-domain cognitive function [13]. A meta-analysis, including studies utilizing CANTAB in MDD patients, revealed deficits in executive function, attention, and memory when compared to healthy controls [14]. Moreover, these cognitive deficits may extend beyond acute episodes, as impairments in executive function, attention, and memory may persist beyond these events [15]. Recent research has increasingly focused on the cognitive deficits observed in MDD, suggesting that these cognitive impairments may be more than state markers of MDD [15].

Previous studies have indicated an association between cognitive function and social function, though reports regarding the specific domains of cognitive function and their relationship with social function are inconsistent [16]. A systematic review of ten studies indicated that impairment in one or more cognitive domains, such as executive function, attention, psychomotor speed, and memory was related to social functioning [17]. However, no single cognitive domain has been consistently reported as a unique predictor [18]. Moreover, most previous studies focused on the relationship between cognitive and social function during the acute phase of depression, with less attention paid to the relationship between cognition and long-term functional outcomes [16–18]. Furthermore, earlier longitudinal studies may have been influenced by the presence of symptoms when analyzing functional outcomes, as they did not rigorously include patients during symptom remission [16–19]. Consequently, the existing evidence does not provide a clear understanding of the relationship between cognitive function and overall functional outcome, potentially leading to a lack of targeted early interventions.

In this study, we aimed to investigate the social function of patients with MDD during symptom remission and explore the cognitive differences between patients exhibiting social dysfunction and those without such impairment. Furthermore, we explored the foundational cognitive domains at the onset of MDD that could predict social dysfunctions during clinical remission.

## Materials and methods

### Participants and study design

We recruited individuals diagnosed with MDD according to the Diagnostic and Statistical Manual of Mental Disorders IV (DSM-IV) diagnostic criteria from the Mental Health Center of West China Hospital, Sichuan University. All participants were interviewed and diagnosed by trained psychiatrists using the Structured Clinical Interview for DSM-IV at baseline assessment. Exclusion criteria included: (a) any medical condition impacting neuropsychological performance, such as neurogenic diseases, endocrine diseases, or metabolic disorders; (b) the presence of other DSM-IV Axis I and II diseases, such as schizophrenia, bipolar disorder, or a history of substance abuse; and (c) having received treatment involving hormone medication or electroconvulsive therapy.

Participants were interviewed at both baseline and follow-up. At the time of follow-up, we comprehensively documented all treatment modalities administered to the patients, encompassing medical therapy, psychotherapy, and physical therapy interventions (such as repetitive transcranial magnetic stimulation therapy) but some patients encountered difficulties in recalling details. Finally, 103 participants were included based on achieving symptomatic remission during the follow-up period. All participants received pharmacological treatment according to guidelines for the management of MDD, while very few of them received additional systematic psychotherapy or physical therapy, presumably due to economic constraints. The flow chart is shown in Supplementary materials Figure S1.

### Demographic, clinical and functional data

At the baseline interview, demographic and clinical information, including age, gender, years of schooling, and first-episode or recurrent MDD were collected. Depression severity was assessed using the 17-item Hamilton Depression Rating Scale (HAMD) [20].

Symptoms and functioning were assessed during follow-up interviews. We only included individuals that were in clinical remission at follow-up (HAMD score ≤ 7 for more than two weeks) [19]. Functional outcome was measured using the Social Disability Screening Schedule (SDSS), a 10-item instrument for assessing the social disability of patients [21], which was derived from the Disability Assessment Schedule [22]. The total scores of patients were confirmed by trained psychiatrists through the interview with patients and their family. The SDSS, widely used in China, demonstrates robust reliability and validity [23]. Total scores were calculated, with higher scores indicating worse social functioning, and a threshold of ≥ 2 points representing functional impairment.

### Cognitive assessment

Cognitive assessment was conducted using three tests of CANTAB, which are standard, computerized, nonlinguistic, and culturally blind tests: rapid visual processing (RVP), pattern recognition memory (PRM), and intra-extra dimensional set shift (IED). These tests were administered to assess three fundamental domains of cognition: attention, memory, and executive function. The selection of outcome measures was based on findings from previous studies [14, 24–31] (Table 1).

**Table 1:** Cognitive tests and measures

Domains and tests	Included variables	Abbreviations
Executive function: IED (Intra-Extra Dimensional Set Shift)	IED stages completed	IED_SC^a^
	IED total errors (adjusted)	IED_TE_A^b^
	IED completed stage errors	IED_CSE^b^
	IED total trials (adjusted)	IED_TT_A^b^
	IED completed stage trials	IED_CST^b^
Visual memory: PRM (Pattern Recognition Memory)	PRM mean latency of correct responses during the immediate mode	PRM_MCLi^b^
	PRM correct responses during the immediate mode	PRM_NCi^a^
	PRM mean latency of correct responses during the delay mode	PRM_MCLd^b^
	PRM correct responses during the delay mode	PRM_NCd^a^
Attention: RVP (Rapid Visual Information Processing)	RVP sensitivity to the target	RVP_A_PR^c^
	RVP tendency to respond	RVP_B_DP^d^
	RVP mean response latency	RVP_ML^b^

Note. ^a^Denotes higher is better. ^b^Denotes lower is better. ^c^Denotes range 0.00 to 1.00, bad to good. ^d^Denotes range − 1.00 to + 1.00, the tendency to respond regardless of whether the target sequence is present

#### RVP

The RVP task was used to assess sustained attention. During this test, a white box was displayed on the computer monitor, inside which digits from two to nine are presented. The digits were shown in a pseudo-random order at a rate of 100 digits per min. Participants were instructed to respond to target sequences of digits (e.g., 2-4-6, 3-5-7, 4-6-8).

#### PRM

The PRM was used to assess the visual recognition memory. During the test, subjects were presented with one set of geometric patterns and then instructed to recognize them when shown with distractors. These geometric patterns were designed such that they could not easily be given verbal labels. This process was repeated using another set of patterns. The second recognition phase was performed after a 20–30 min delay.

#### IED

The IED test was used to measure cognitive flexibility. This is an essential part of executive function, which is dominated by the prefrontal lobe [32]. In this task, participants use feedback to deduce a rule that determines which stimulus is correct. Initially, stimuli varied along only one dimension (e.g., shape). The stimuli and/or rules changed after six correct responses. Participants had to switch attention to a new stimulus from the same stimulus dimension (e.g., shape, intra-dimensional shift) as well as a new stimulus from a different stimulus dimension (e.g., lines, extra-dimensional shift).

### Data analyses

All analyses were performed using R (R version R 4.0.2, R Studio version 1.1.463) (https://cran.r-project.org). Descriptive statistics was conducted by mean and SD for continuous variables, and by number (n) for categorical variables. Participants were dichotomized into normal social function group and poor social function group according to the SDSS total scores. The chi-square test and two-tailed independent t-test were used to compare differences in sociodemographic, clinical, and neuropsychological variables between groups. A logistic regression analysis was performed to identify specific factors associated with functioning outcomes. Independent variables in the regression model were selected based on the previous steps with variables that showed statistical significance. The dependent variable in the regression model determined the presence of impaired functioning (according to the SDSS score at follow-up). Multiple imputations were used to handle three missing data in the first-episode or recurrent and one missing data in the RVP mean response latency. The missing data were due to recording errors. Multiple imputations were conducted using the R package “mice”. Two-sided tests with the threshold for statistical significance set at *p* < 0.05.

## Results

### Sociodemographic and clinical characteristics

The sociodemographic and clinical characteristics of the respondents are presented in Table 2. A total of 103 patients (35 male and 68 female) were included in the study. The mean age was 27.53 ± 9.7 years, and the mean number of years of schooling was 13.71 ± 3.08 years. Sixty-five out of the 103 participants had first-episode depression, while thirty-eight had recurrent depression. The mean HAMD score at baseline was 20.65 ± 4.89, and the mean HAMD score at follow-up was 2.42 ± 2.37. The mean follow-up time was 42.92 ± 22.87 months. No significant differences were observed between groups in terms of clinical and sociodemographic variables (Table 2).

**Table 2:** The sociodemographic and clinical variables between two groups

	Overall (*n* = 103)	Normal social function group (*n* = 81)	Poor social function group (*n* = 22)	*p*-value^a^
Age	27.53 ± 9.70	26.91 ± 9.62	29.82 ± 9.88	0.214
First-episode or recurrent	58/45	44/37	14/8	0.590
Gender (Male/Female)	35/68	26/55	9/13	0.603
Years of schooling	13.71 ± 3.08	13.74 ± 2.99	13.59 ± 3.45	0.841
HAMD (Baseline)	20.65 ± 4.89	20.53 ± 4.58	21.10 ± 6.00	0.641
HAMD (Follow-up)	2.42 ± 2.37	2.46 ± 2.40	2.27 ± 2.27	0.748
Follow-up time	42.92 ± 22.87	42.40 ± 23.33	44.86 ± 21.47	0.656

Note. ^a^*p*-values obtained via the t-test for continuous variables and Chi-Square test for binary variables. The normal social function group represent SDSS total scores less than 2. The poor social function group represent SDSS total score greater than or equal to 2

HAMD = Hamilton Depression Rating Scale

### Group differences on cognitive test performance

The cognitive variables at the baseline of two groups are described in Table 3. At the baseline, when compared to the group with normal social function, patients in the poor social function group (*n* = 22) showed worse cognitive function in the visual recognition memory and executive function tests. Specifically, the group displayed fewer correct responses in the immediate mode of the PRM test (PRM_NCi) and more IED completed stage errors (IED_CSE) compared to the normal social function group (*n* = 81). However, no significant differences were observed between the groups regarding other cognitive variables.

**Table 3:** Comparison of cognitive performance between two groups at baseline

	Overall (*n* = 103)	Normal social function group (*n* = 81)	Poor social function group (*n* = 22)	*p*-value
IED_SC	8.43 ± 1.20	8.35 ± 1.31	8.73 ± 0.63	0.188
IED_TE_A	29.40 ± 29.04	30.49 ± 31.58	25.36 ± 16.60	0.465
IED_CSE	14.84 ± 9.99	13.81 ± 9.35	18.64 ± 11.48	**0.044**
IED_TT_A	104.17 ± 50.41	106.06 ± 54.54	97.23 ± 30.76	0.469
IED_CST	75.53 ± 21.99	73.35 ± 21.36	83.59 ± 22.91	0.052
PRM_MCLi	2112.77 ± 649.24	2065.80 ± 598.57	2285.68 ± 801.63	0.160
PRM_NCi	11.24 ± 0.95	11.36 ± 0.86	10.82 ± 1.18	**0.018**
PRM_MCLd	2111.72 ± 696.15	2067.94 ± 619.35	2272.9 ± 926.26	0.222
PRM_NCd	8.93 ± 2.02	8.94 ± 2.04	8.91 ± 1.97	0.952
RVP_A_PR	0.89 ± 0.08	0.89 ± 0.09	0.90 ± 0.06	0.693
RVP_B_DP	0.92 ± 0.15	0.92 ± 0.08	0.89 ± 0.30	0.380
RVP_ML	423.29 ± 96.59	423.45 ± 102.11	422.74 ± 74.75	0.980

Note. Data presented as means ± standard deviations; Significant differences (*p* < 0.05) are marked in bold

IED = Intra-extra dimensional set shift, RVP = Rapid visual processing, PRM = Pattern recognition memory

IED_SC = IED Stages completed, IED_TE_A = IED Total errors (adjusted), IED_CSE = IED Completed stage errors, IED_TT_A = IED Total errors (adjusted), IED_CST = IED Completed stage trials; PRM_MCLi = PRM Mean correct latency (immediate), PRM_NCi = PRM Number correct (immediate), PRM_MCLd = PRM Mean correct latency (delayed), PRM_NCd = PRM Number correct (delayed); RVP_A_PR = RVP A’, RVP_B_DP = RVP B’, RVP_ML = RVP Mean latency

### Predictors of social function during symptomatic remission

**Fig. 1:**
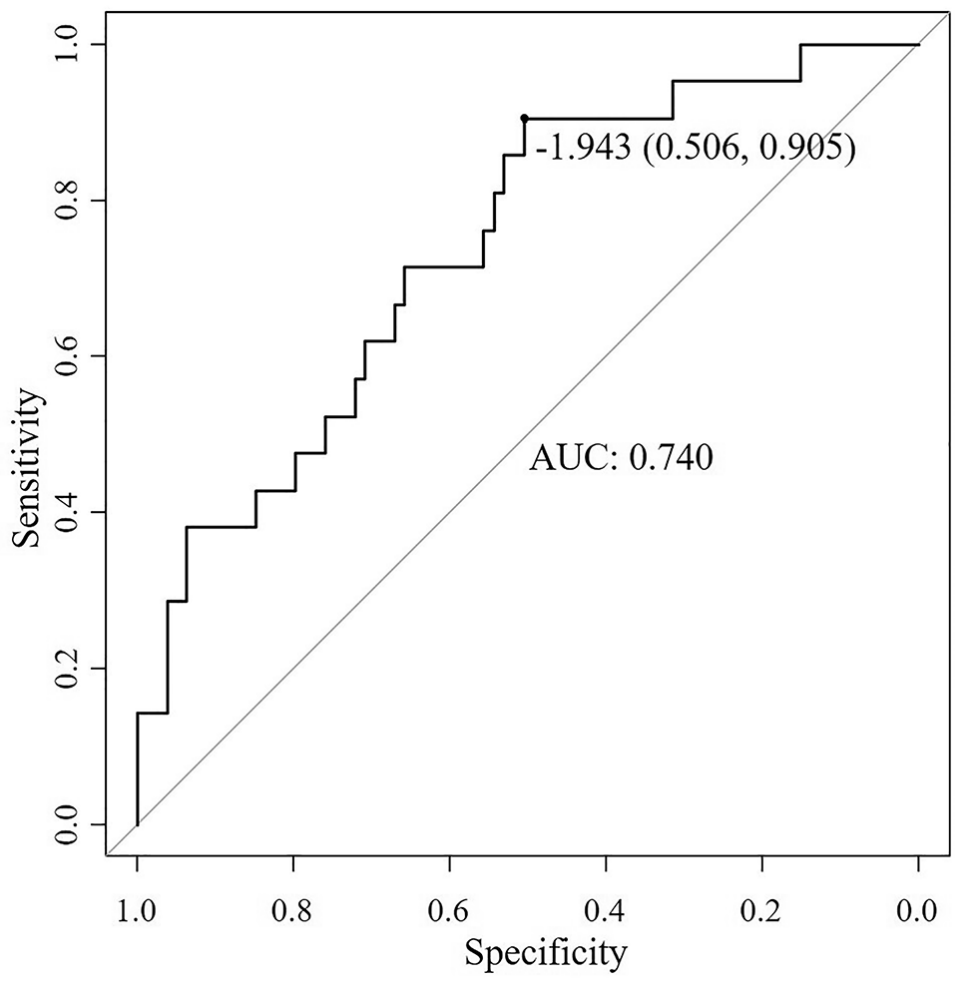
ROC curve of the social function during symptomatic remission ROC = receiver operating characteristic, AUC = area under curve

A logistic regression model was constructed to investigate the predictive ability of various baseline variables on functioning during the remission stage. Only variables with a significance level of *p* < 0.05 were included in the model. Moreover, factors such as age, gender, years of schooling, HAMD scores at baseline and follow-up, follow-up duration, and first-episode/recurrent depression were also included as they are relevant factors that could potentially impact both cognitive function and social function (Table 4). The model’s AUC value was 0.740 (95% CI: 0.625–0.855; Fig. 1).

**Table 4:** The logistic regression analysis result of social function in remission and five factors

Characteristic	OR	95% CI	*p*-value
(Intercept)	315.54	0.05, 2,313,888	0.195
PRM_NCi	0.55	0.31, 0.93	**0.030**
IED_CSE	1.07	1.01, 1.13	**0.014**
Age	1.02	0.96, 1.08	0.494
Gender	0.41	0.13, 1.27	0.124
HAMD (Baseline)	1.03	0.91, 1.16	0.635
HAMD (Follow-up)	1.00	0.50, 2.09	0.992
Follow-up time	1.00	0.93, 1.08	0.989
First-episode/recurrent depression	0.36	0.16, 1.62	0.123
Years of schooling	1.01	0.55, 2.20	0.908

Note. Bold values represent statistically significant results

## Discussion

Our study found that about 1/5 of participants remained socially dysfunctional during symptomatic remission according to their scores of SDSS. Participants with poorer social function performed worse on tests of visual memory and executive function. Cognitive performance in visual memory and executive function in the acute phase may be important predictors of social function during symptomatic remission in patients with major depressive disorder (MDD).

In this study, 22 of the 103 participants experienced impaired social function during clinical remission. This finding aligns with limited studies on long-term functional outcomes in MDD [12, 33], which have indicated that a significant number of MDD patients still suffered from social dysfunction even in clinical remission. This result highlights the importance of prioritizing functional remission as the primary goal of MDD treatment.

The relationship between baseline cognition and functional outcomes in MDD has been unexplored. In a prospective study, cognitive performance and functional outcome were evaluated at admission, 3 months after remission (defines as an improvement of at least 50% from admission Hamilton Depression Rating Scale score and no longer meeting syndromal criteria) and discharge. This study suggested that poorer executive function may be a marker of poorer functional outcome in MDD [34]. Another earlier prospective study reported an association between baseline cognitive deficits in the domains of attention, memory, and executive function and functional outcome at 6 months [35]. A previous review indicated that cognitive deficits in memory, attention, and executive function appear to be mediators of impairment in vocational function [36]. Our results show that patients with functional impairment during symptomatic remission performed worse in visual memory and executive function at baseline, which is consistent with these previous studies. Our study included participants who achieved symptomatic remission (HAMD score ≤ 7 for more than two weeks) at follow-up in order to minimize the effects of residual symptoms. In addition, our study utilized the Cambridge Neuropsychological Test Automated Battery (CANTAB), a highly precise and objective battery in cognitive assessments. Consequently, our study provides more compelling evidence for the predictive role of cognitive function in social function during symptom remission. As cognitive impairments have been demonstrated to persist in MDD, our findings may help identify patients who are at risk for poor functional outcomes using measures of cognitive function during the acute phase, thus allowing for early intervention.

We also found that certain neurocognitive domains at the baseline, such as visual memory and executive function, may be important potential predictors of social function during symptomatic remission in patients with major depressive disorder (MDD). Visual memory may contribute to several aspects of social functioning, including financial skills and driving performance [37, 38]. Impairments in visual memory increase the likelihood of forgetting important information, with potentially significant negative consequence as most information in our life is conveyed visually. Executive function comprises of a complex system of skills, including behavioral inhibition, mental flexibility, and working memory, all of them play vital roles in daily life [39, 40]. These executive functions contribute to several aspects of social functioning. For example, cognitive flexibility is crucial for MDD patients to meeting the demands of society, requiring rapid and flexible adjustments of behavior [34]. Cognitive flexibility also helps individuals switch attention between different conditions, such as work, study or housework. On the contrary, cognitive inflexibility may lead to inappropriate emotional reactions that negatively impact social interactions [41]. Therefore, visual memory and executive function emerge as potential early intervention targets for improving functional outcomes in MDD.

Our results extend the current understanding of the relationship between baseline cognition and functional outcomes, especially in young patients with MDD. However, caution is warranted due to the sample size and the consideration of multiple comparisons. Besides, the structured performance rating method of measuring social functioning may lead to bias, because the evaluators may not have enough information to arrive at a valid rating. Moreover, different cognitive domains could have varying impacts on various types of occupations. Further longitudinal studies with large sample sizes, as well as studies considering the types of jobs and employment status are warranted to confirm our findings. And the limited number of neuropsychological tests may lead to ceiling effects, more comprehensive measures of cognitive function may reveal more differences. Additionally, the influence of clinical factors such as age at onset, duration of illness, history of depressive episodes and number of psychiatric hospitalizations wasn’t analyzed in our study. Future research should collect comprehensive clinical information to gain a deeper understanding of the complex relationships between clinical variables and cognitive and social functioning, ultimately leading to more targeted and effective treatment strategies.

## Conclusions

Even in clinical remission, part of MDD patients still suffered from social dysfunction. Better visual memory and executive function in the acute phase of depression may contribute to better social functioning during symptomatic remission in MDD. Early interventions targeting executive function and visual memory in people with MDD may have a positive impact on functional recovery.

### Electronic supplementary material

Below is the link to the electronic supplementary material.

Supplementary Material 1

## Data Availability

The datasets used and analyzed during the current study are available from the corresponding author on reasonable request.

## Abbreviations

MDD: Major depressive disorder
CANTAB: Cambridge Neuropsychological Test Automated Battery
DSM-IV: Diagnostic and Statistical Manual of Mental Disorders, fourth edition
HAMD: 17-item Hamilton Depression Rating Scale
SDSS: Social Disability Screening Schedule
RVP: Rapid visual processing
PRM: Pattern recognition memory
IED: Intra-extra dimensional set shift
