# Supplementary figures and images for: Relationship between cognitive function and functional outcomes in remitted major depression
Source: BMC Psychiatry. 2024 Apr 24;24:311. doi: 10.1186/s12888-024-05675-6 (PMC11040809; doi:10.1186/s12888-024-05675-6)

## Supplementary Materials:


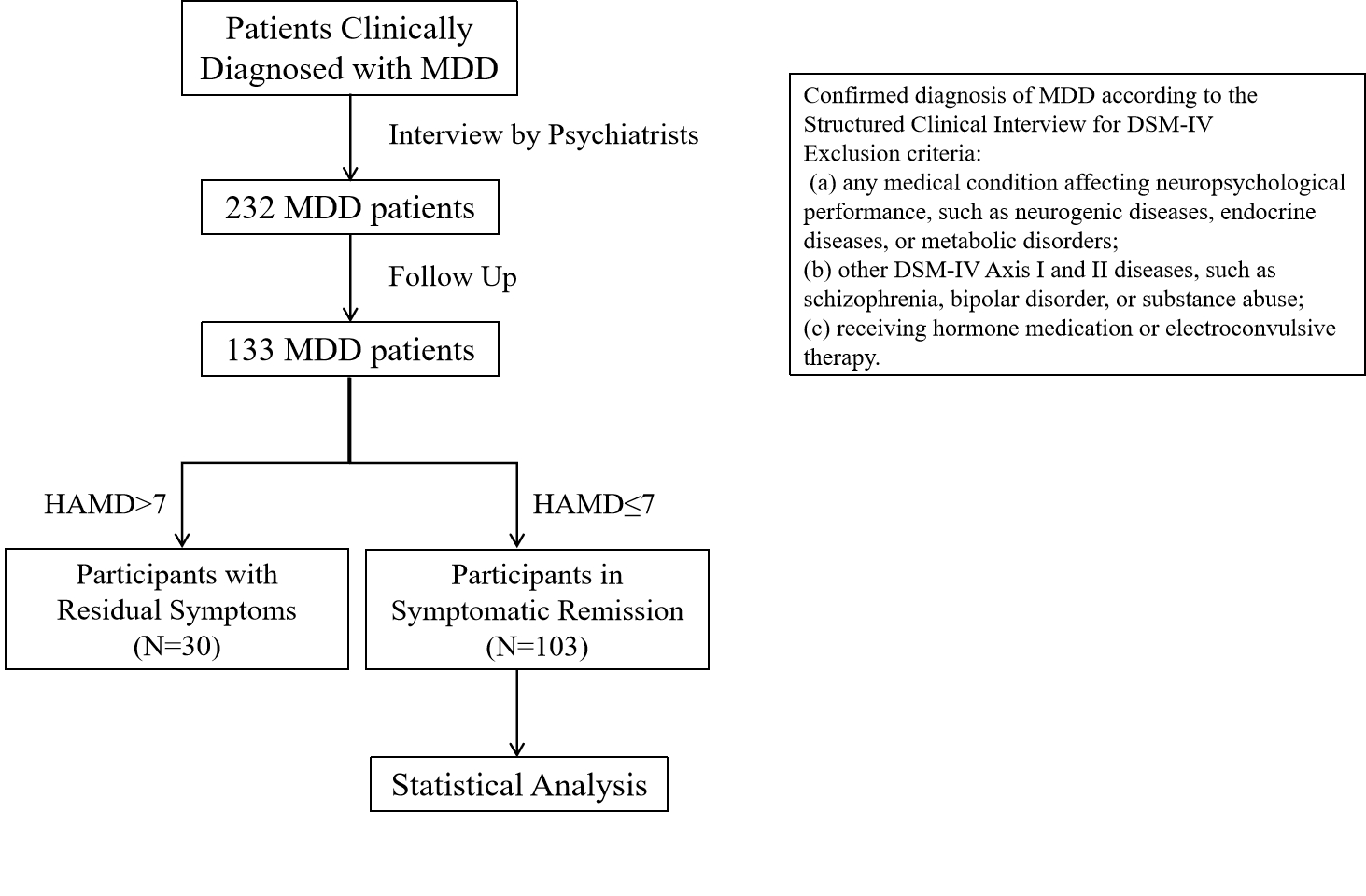


**Figure S1.** Flowchart of the study.

**Note:** MDD, major depressive disorder.

Supplement: Supplementary file 1 — Supplementary Material 1 [file 12888_2024_5675_MOESM1_ESM.docx]
